# BUILDING COHESIVE CONVOYS: IMPROVING STABILITY AND PREPAREDNESS OF DIRECT CARE WORKERS IN ASSISTED LIVING

**DOI:** 10.1093/geroni/igz038.1658

**Published:** 2019-11-08

**Authors:** Jennifer C Morgan, Elisabeth Burgess, Christina Barmon, Candace L Kemp

**Affiliations:** 1 Georgia State University, Atlanta, Georgia, United States; 2 Central Connecticut State University, New Britain, Connecticut, United States

## Abstract

Person-centered collaborative care arrangements that empower residents, families, and care partners require supports for resident and their “care convoys”—the evolving collection of individuals who provide formal and informal care. Direct care workers (DCWs) are essential in supporting resident needs within the complex and dynamic environment of assisted living. The stability and preparedness of this workforce is central to improving quality of life for residents. This paper identifies key factors influencing the integration of DCWs in the convoys and explore supportive employment practices to strengthen the convoy. This analysis uses data from a 5-year mixed-method qualitative study of eight assisted living communities. Time pressures, AL policies and practices, work overload, lack of training, and turnover impact whether direct care workers are empowered as full members of the care convoy. Supportive employment practices (e.g. training, onboarding, career opportunities, rewards and recognition) are discussed as potential solutions for building consensus and collaboration.

